# Supplementary material for: Study of susceptibility to antibiotics and molecular characterization of high virulence Staphylococcus aureus strains isolated from a rural hospital in Ethiopia
Source: PLoS One. 2020 Mar 12;15(3):e0230031. doi: 10.1371/journal.pone.0230031 (PMC7067403; doi:10.1371/journal.pone.0230031)

S3 Figure. Alignment of sequences of fragment obtain from multiplex PCR for SCC*mec* typing in strain 73 (Subject) and AB063172 from database GenBank as reference of subtyping IVa of SCC*mec* typing (Query). Similarity of 100% between both sequences proves that strain 73 present IVa SCC*mec* subtype (59).


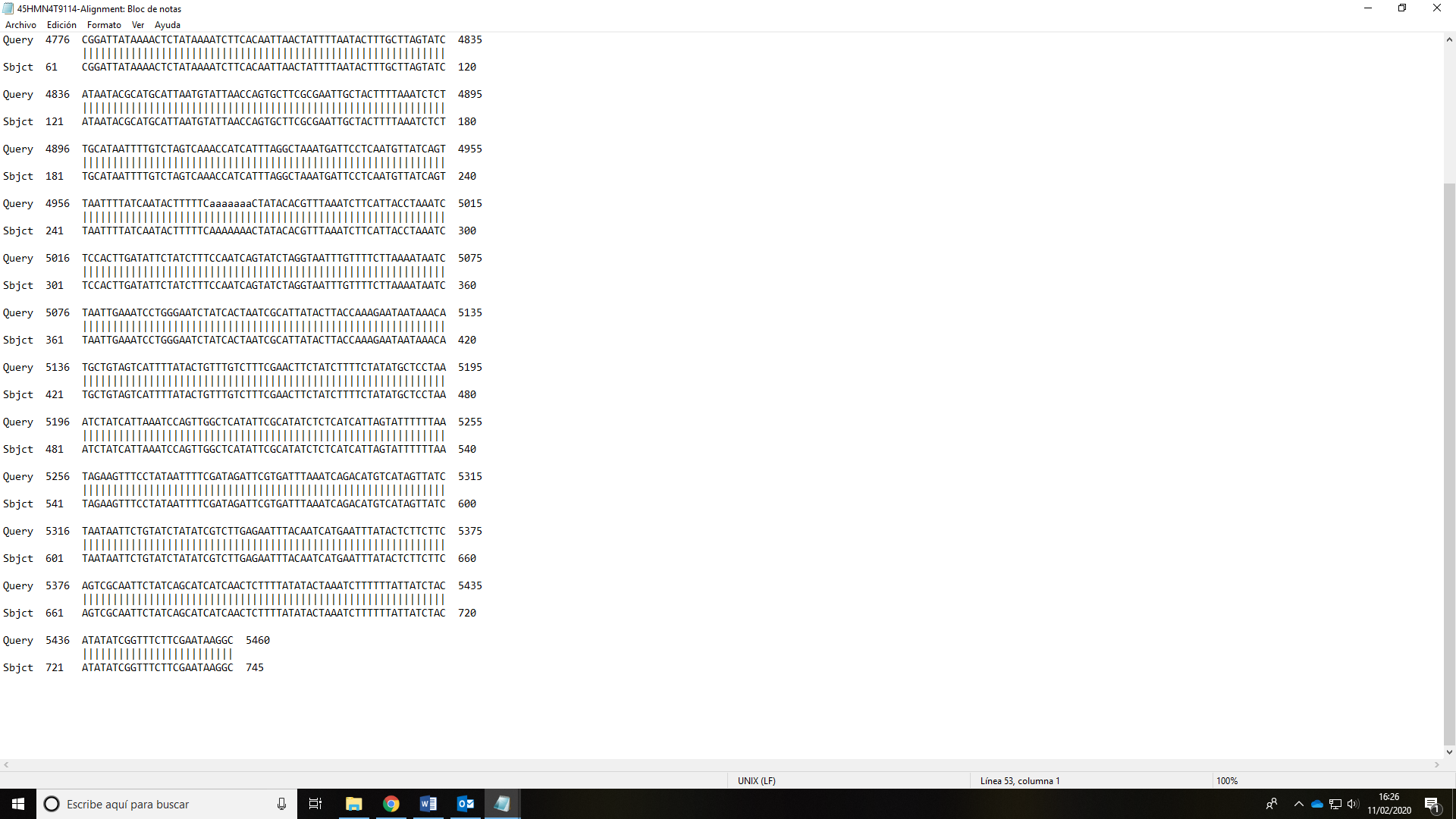

Supplement: S1 Fig — Similarity of 100% between both sequences proves that strain 73 present IVa SCCmec subtype (59). (DOCX) [file pone.0230031.s001.docx]
