# Supplementary material for: Study of susceptibility to antibiotics and molecular characterization of high virulence Staphylococcus aureus strains isolated from a rural hospital in Ethiopia
Source: PLoS One. 2020 Mar 12;15(3):e0230031. doi: 10.1371/journal.pone.0230031 (PMC7067403; doi:10.1371/journal.pone.0230031)
Supplement: S2 Fig — (DOCX) [file pone.0230031.s002.docx]

S4 Figure. Results of statistical correlational analysis presented virulence genes and MLST STs highly dispersed, showing that there was no relationship between these two factors.
