# Supplementary material for: Study of susceptibility to antibiotics and molecular characterization of high virulence Staphylococcus aureus strains isolated from a rural hospital in Ethiopia
Source: PLoS One. 2020 Mar 12;15(3):e0230031. doi: 10.1371/journal.pone.0230031 (PMC7067403; doi:10.1371/journal.pone.0230031)
Supplement: S1 Table — The PCR reaction mixture was as follows: 10 μl of commercial preparation NZYTaq II 2× Green Master Mix (NZYTech, MB358), 2 μl of primer mixture, 13 μl of DNA-free sterile water and 2 μl of genomic DNA; the final reaction volume was 25 μl. (DOCX) [file pone.0230031.s003.docx]

**Supporting information**

**S1 Table.** Primers and PCR conditions for amplification of different genes. The PCR reaction mixture was as follows: 10 μl of commercial preparation NZYTaq II 2× Green Master Mix (NZYTech, MB358), 2 μl of primer mixture, 13 μl of DNA-free sterile water and 2 μl of genomic DNA; the final reaction volume was 25 μl.

| **Gene** | **Product** | **Primer sequence (5´-3´)** | **Annealing temperature (°C)** | **Amplicon length (pb)** | **Reference** |
| --- | --- | --- | --- | --- | --- |
| ***DNAr 16S*** | ARN ribosomal 16S | Fw: AGAGTTTGATCMTGGCTCAG  Rv: TACGGYTACCTTGTTACGACTT | 55 | 1.465 | Lane, 1991 |
| ***arc*** | Carbamate kinase | Fw: TTGATTCACCAGCGCGTATTGTC  Rv: AGGTATCTGCTTCAATCAGCG | 55 | 456 | MLST website |
| ***aro*** | Shikimate dehydrogenase | Fw: ATCGGAAATCCTATTTCACATTC  Rv: GGTGTTGTATTAATAACGATATC | 55 | 456 | MLST website |
| ***glp*** | Glycerol kinase | Fw: CTAGGAACTGCAATCTTAATCC  Rv: TGGTAAAATCGCATGTCCAATTC | 55 | 465 | MLST website |
| ***gmk*** | Guanylate kinase | Fw: ATCGTTTTATCGGGACCATC  Rv: TCATTAACTACAACGTAATCGTA | 55 | 417 | MLST website |
| ***pta*** | Phosphate acetyltransferase | Fw: GTTAAAATCGTATTACCTGAAGG  Rv: GACCCTTTTGTTGAAAAGCTTAA | 55 | 474 | MLST website |
| ***tpi*** | Triosephosphate isomerase | Fw: TCGTTCATTCTGAACGTCGTGAA  Rv: TTTGCACCTTCTAACAATTGTAC | 55 | 402 | MLST website |
| ***yqi*** | Acetyle coenzyme A acetyltransferase | Fw: CAGCATACAGGACACCTATTGGC  Rv: CGTTGAGGAATCGATACTGGAAC | 55 | 516 | MLST website |
| ***hla*** | α-hemolisin | Fw: CTGATTACTATCCAAGAAATTCGATTG  Rv: CTTTCCAGCCTACTTTTTTATCAGT | 58 | 209 | Delgado et al., 2011 |
| ***tstH*** | Shock syndrom toxic toxin | Fw: AAGCCCTTTGTTGCTTGCG  Rv: ATCGAACTTTGGCCCATACTTT | 62 | 404 | Delgado et al., 2011 |
| ***lukPV*** | Leukocidin Panton-Valentine | Fw: ATCATTAGGTAAAATGTCTGGACATGATCCA  Rv: GCATCAASTGTATTGGATAGCAAAAGC | 65 | 433 | Delgado et al., 2011 |
| ***fnbA*** | Fibronectin binding protein A | Fw: CACAACCAGCAAATATAG  Rv: CTGTGTGGTAATCAATGTC | 50 | 1226 | Delgado et al., 2011 |
| ***mecA*** | Meticillin resistance | Fw: GGTCCCATTAACTCTGAAG  Rv: AGTTCTGCAGTACCGGATTTTGC | 57 | 1020 | Delgado et al., 2011 |
