# Supplementary material for: Study of susceptibility to antibiotics and molecular characterization of high virulence Staphylococcus aureus strains isolated from a rural hospital in Ethiopia
Source: PLoS One. 2020 Mar 12;15(3):e0230031. doi: 10.1371/journal.pone.0230031 (PMC7067403; doi:10.1371/journal.pone.0230031)
Supplement: S2 Table — The table shows alleles assigned to each of the seven MLST housekeeping genes (arc, aro, glp, gmk, pta, tpi, yqi) and for each strain (first column), as well as sequence type (“ST” column) assigned according to allele combination. “N” sequence type and alleles are assigned to those which do not correspond to any ST (green) or allele (orange) from the database. New STs were assigned numbers (1 to 37). The only ST in the database characterised in Ethiopia (Et) was included. (DOCX) [file pone.0230031.s004.docx]

**S2 Table.** MLST analysis results. The table shows alleles assigned to each of the seven MLST housekeeping genes (*arc*, *aro*, *glp*, *gmk*, *pta*, *tpi*, *yqi*) and for each strain (first column), as well as sequence type (“ST” column) assigned according to allele combination. “N” sequence type and alleles are assigned to those which do not correspond to any ST (green) or allele (orange) from the database. New STs were assigned numbers (1 to 37). The only ST in the database characterised in Ethiopia (Et) was included.

| **Strains** | ***arc*** | ***aro*** | ***glp*** | ***gmk*** | ***pta*** | ***tpi*** | ***yqi*** | **ST** |
| --- | --- | --- | --- | --- | --- | --- | --- | --- |
| **Et** | 1 | 37 | 48 | 19 | 96 | 26 | 39 | 727 |
| **1** | 177 | 4 | 1 | 4 | 12 | 41 | 10 | N1 |
| **2** | 6 | 5 | 6 | 2 | 7 | 14 | 5 | 121 |
| **3** | 6 | 5 | 6 | 2 | 7 | 14 | 5 | 121 |
| **4** | 6 | 22 | 6 | 2 | 7 | 14 | 10 | N2 |
| **5** | 1 | 1 | 1 | 1 | 22 | 1 | 1 | 772 |
| **6** | 201 | 348 | 236 | 66 | 82 | 267 | 269 | 2431 |
| **7** | 2 | 4 | N | 3 | 144 | 3 | 2 | N3 |
| **8** | 1 | 4 | 1 | 4 | 12 | 1 | 10 | 5 |
| **9** | 13 | 4 | 1 | 4 | 613 | 457 | 10 | 328 |
| **10** | 1 | 727 | 1 | 4 | 12 | 1 | 10 | 4997 |
| **11** | 13 | 13 | 1 | 1 | 12 | 11 | 13 | 15 |
| **12** | 6 | 5 | 6 | 2 | 7 | 14 | 655 | N4 |
| **15** | 6 | 5 | 6 | 2 | 7 | 14 | 5 | 121 |
| **16** | 13 | 13 | 1 | 1 | 12 | 11 | 13 | 15 |
| **17** | N | 31 | 1 | 4 | 12 | 1 | 10 | N5 |
| **18** | 13 | 13 | 1 | 1 | 12 | 11 | 13 | 15 |
| **19** | 46 | 75 | 49 | 44 | 13 | 68 | 60 | 152 |
| **20** | 13 | 13 | 1 | 1 | 12 | 11 | 13 | 15 |
| **21** | 46 | 75 | 49 | 44 | 13 | 68 | 60 | 152 |
| **22** | N | 4 | 1 | 4 | 12 | 1 | 10 | N6 |
| **23** | 22 | 1 | 14 | 23 | 12 | 537 | 31 | N7 |
| **24** | 6 | 1 | 298 | 44 | 289 | 291 | N | N8 |
| **25** | 1 | 5 | 6 | 2 | 7 | 14 | 78 | N9 |
| **26** | 10 | 349 | 6 | 2 | 260 | 58 | 2 | N10 |
| **27** | 46 | 75 | 49 | 44 | 13 | 68 | 60 | 152 |
| **28** | 13 | 13 | 1 | 279 | 12 | 11 | 13 | N11 |
| **29** | 13 | 13 | 1 | 279 | 12 | 11 | 13 | N11 |
| **30** | 6 | 5 | 6 | 2 | 7 | 14 | 37 | N12 |
| **31** | 46 | 75 | 212 | 44 | 13 | 68 | 60 | 1633 |
| **32** | 46 | 75 | 49 | 44 | 13 | 68 | 60 | 152 |
| **33** | 46 | 75 | 49 | 44 | 13 | 68 | 60 | 152 |
| **34** | 13 | 13 | 299 | 279 | 12 | 34 | 13 | N13 |
| **35** | 354 | 256 | 358 | 281 | 221 | 302 | 13 | N14 |
| **36** | 1 | 1 | 1 | 78 | 22 | 457 | 656 | N15 |
| **37** | 1 | 1 | 1 | 279 | 67 | 486 | 656 | N16 |
| **38** | 1 | 4 | 1 | 4 | 86 | 495 | 10 | N17 |
| **39** | 6 | 5 | 6 | 281 | 7 | 14 | 94 | N18 |
| **40** | 6 | 5 | 6 | 281 | 7 | 14 | 510 | N19 |
| **41** | 1 | 4 | 1 | 4 | 12 | 1 | 256 | N20 |
| **42** | 46 | 75 | 49 | 44 | 13 | 68 | 60 | 152 |
| **43** | 2 | 518 | 2 | 281 | 6 | 3 | 500 | N21 |
| **44** | 6 | 1 | 1 | 279 | 22 | 457 | 541 | N22 |
| **45** | 6 | 55 | 45 | 98 | 109 | 219 | 477 | N23 |
| **46** | 6 | 5 | 6 | 281 | 7 | 14 | 94 | N18 |
| **47** | 1 | 4 | 1 | 4 | 12 | 1 | 80 | 676 |
| **48** | 410 | 4 | 1 | 4 | 12 | 1 | 10 | 3887 |
| **50** | 13 | 643 | 1 | 279 | 12 | 11 | 13 | N24 |
| **51** | 22 | 1 | 14 | 23 | 12 | 4 | 398 | 3199 |
| **52** | 354 | 256 | 358 | 66 | 221 | 302 | 328 | 3224 |
| **53** | 6 | 5 | 6 | 281 | 7 | 14 | 60 | N25 |
| **54** | 354 | N | 236 | 169 | 194 | 411 | N | N26 |
| **55** | 46 | 75 | 49 | 44 | 13 | 68 | 60 | 152 |
| **56** | 6 | 5 | 6 | 281 | 7 | 14 | 5 | N27 |
| **57** | 13 | 13 | 299 | 279 | 12 | 11 | 13 | N28 |
| **58** | 189 | 13 | 1 | 1 | 12 | 11 | 13 | 1698 |
| **59** | 6 | 5 | 6 | 281 | 7 | 14 | 5 | N27 |
| **61** | 565 | 13 | 1 | 279 | 12 | 11 | 13 | N29 |
| **62** | 7 | 6 | 1 | 8 | 8 | 8 | 6 | N30 |
| **63** | 10 | 349 | 6 | 260 | 260 | 58 | 462 | N31 |
| **64** | 1 | 4 | 1 | 12 | 12 | 1 | 10 | N32 |
| **65** | 13 | 13 | 1 | 279 | 12 | 11 | 13 | N11 |
| **66** | 46 | 75 | 49 | 44 | 13 | 68 | 60 | 152 |
| **67** | 46 | 75 | 49 | 44 | 13 | 68 | 60 | 152 |
| **68** | 22 | 1 | 14 | 23 | 12 | 4 | 233 | 2066 |
| **69** | 1 | 4 | 1 | 4 | 12 | 1 | 10 | 5 |
| **70** | 46 | 75 | 49 | 44 | 13 | 68 | 60 | 152 |
| **71** | 46 | 75 | 49 | 44 | 13 | 68 | 60 | 152 |
| **72** | 46 | 75 | 49 | 44 | 13 | 68 | 60 | 152 |
| **73** | 445 | 3 | 549 | 279 | 64 | 497 | 306 | N33 |
| **74** | 6 | 5 | 6 | 281 | 7 | 14 | 5 | N27 |
| **75** | N | N | 236 | 66 | N | 219 | 477 | N34 |
| **76** | 46 | 75 | 49 | 44 | 13 | 68 | 60 | 152 |
| **77** | 46 | 75 | 49 | 44 | 13 | 68 | 60 | 152 |
| **78** | 6 | 5 | 6 | 281 | 7 | 48 | 5 | N35 |
| **79** | 22 | 1 | 14 | 23 | 12 | 497 | 31 | N36 |
| **80** | 1 | 4 | 1 | 4 | 12 | 457 | 10 | N37 |
| **81** | 46 | 75 | 49 | 44 | 13 | 68 | 60 | 152 |
| **82** | 354 | 256 | 358 | 66 | 221 | 302 | 328 | 3224 |
| **83** | 13 | 13 | 1 | 279 | 12 | 11 | 13 | N11 |
| **84** | 22 | 1 | 14 | 23 | 12 | 497 | 31 | N36 |
