# Supplementary material for: Study of susceptibility to antibiotics and molecular characterization of high virulence Staphylococcus aureus strains isolated from a rural hospital in Ethiopia
Source: PLoS One. 2020 Mar 12;15(3):e0230031. doi: 10.1371/journal.pone.0230031 (PMC7067403; doi:10.1371/journal.pone.0230031)
Supplement: Attachment — Submitted filename: PONE-D-19-29715_reviewer.pdf [file pone.0230031.s005.pdf]

PLOS ONE

Study of susceptibility to antibiotics and molecular characterisation of high virulence Staphylococcus aureus strains isolated from a rural hospital in Ethiopia

--Manuscript Draft--

**Manuscript Number:**	PONE-D-19-29715
**Article Type:**	Research Article
**Full Title:**	Study of susceptibility to antibiotics and molecular characterisation of high virulence Staphylococcus aureus strains isolated from a rural hospital in Ethiopia
**Short Title:**	Susceptibility to antibiotics and molecular characterisation of Staphylococcus aureus strains from Ethiopia
**Corresponding Author:**	Cristina Verdú-Expósito<br>University of Alcala<br>Alcalá de Henares, SPAIN
**Keywords:**	Staphylococcus aureus; Ethiopia; MLST; resistance; phylogeny; virulence
**Abstract:**	We characterised 80 Staphylococcus aureus strains isolated from human patients with SSTIs at a rural hospital in Ethiopia.<br>Susceptibility to antibiotic of all strains was tested. The MLST method was used to type and a phylogenetic analysis was conducted employing the sequences of 7 housekeeping genes. PCR amplification was used to investigate the presence of the following virulence genes in all strains: hla (α-haemolysin), tstH (toxic shock syndrome toxin), luk PV (Panton-Valentine leukocidin), fnbA (fibronectin binding protein A) and mecA (methicillin resistance).<br>Most of the strains were resistant to penicillin and ampicillin, but only 3 strains were resistant to oxacillin, and 1 of them was a true MRSA. The MLST results showed a high diversity of sequence types (ST), 55% of which were new, and ST152 was the most prevalent. A phylogeny study showed that many of the new STs were phylogenetically related to other previously described STs, but bore little relationship to the only ST from Ethiopia described in the database. Virulence gene detection showed a high prevalence of strains encoding the hla, fnbA and pvl genes (98.77%, 96.3% and 72.84%, respectively), a low prevalence of the tst gene (13.58%) and a markedly low prevalence of MRSA (1.25%).<br>S. aureus strains isolated from patients in a rural area in Ethiopia showed low levels of antibiotic resistance, except to penicillin. Moreover, this study reveals new STs in Eastern Africa that are phylogenetically related to other previously described STs, and confirm the high prevalence of the pvl gene and the low prevalence of MRSA on the continent.
**Order of Authors:**	Cristina Verdú-Expósito
	Juan Romanyk
	Juan Cuadros-González
	Abraham Tesfamariam
	José Luis Copa-Patiño
	Jorge Pérez-Serrano
	Juan Soliveri
**Additional Information:**	
**Question**	**Response**
**Financial Disclosure**<br><br>Enter a financial disclosure statement that describes the sources of funding for the work included in this submission. Review	The author(s) received no specific funding for this work.

Powered by Editorial Manager® and ProduXion Manager® from Aries Systems Corporation

the submission guidelines for detailed requirements. View published research articles from *PLOS ONE* for specific examples.<br><br>This statement is required for submission and **will appear in the published article** if the submission is accepted. Please make sure it is accurate.<br><br>**Unfunded studies**<br>Enter: *The author(s) received no specific funding for this work.*<br><br>**Funded studies**<br>Enter a statement with the following details:<br>• Initials of the authors who received each award<br>• Grant numbers awarded to each author<br>• The full name of each funder<br>• URL of each funder website<br>• Did the sponsors or funders play any role in the study design, data collection and analysis, decision to publish, or preparation of the manuscript?<br>• **NO** - Include this sentence at the end of your statement: *The funders had no role in study design, data collection and analysis, decision to publish, or preparation of the manuscript.*<br>• **YES** - Specify the role(s) played.<br><br>* typeset	
**Competing Interests**<br><br>Use the instructions below to enter a competing interest statement for this submission. On behalf of all authors, disclose any competing interests that could be perceived to bias this work—acknowledging all financial support and any other relevant financial or non-financial competing interests.<br><br>This statement **will appear in the published article** if the submission is accepted. Please make sure it is accurate. View published research articles from *PLOS ONE* for specific examples.	The authors have declared that no competing interests exist.

Powered by Editorial Manager® and ProduXion Manager® from Aries Systems Corporation

**NO authors have competing interests**<br><br>Enter: *The authors have declared that no competing interests exist.*<br><br>**Authors with competing interests**<br><br>Enter competing interest details beginning with this statement:<br><br>*I have read the journal's policy and the authors of this manuscript have the following competing interests: [insert competing interests here]*<br><br>* typeset	
**Ethics Statement**<br><br>Enter an ethics statement for this submission. This statement is required if the study involved:<br><br>• Human participants<br>• Human specimens or tissue<br>• Vertebrate animals or cephalopods<br>• Vertebrate embryos or tissues<br>• Field research<br><br>Write "N/A" if the submission does not require an ethics statement.<br><br>General guidance is provided below. Consult the submission guidelines for detailed instructions. **Make sure that all information entered here is included in the Methods section of the manuscript.**	N/A<br>After asking to the hospital, the study didn't require any ethics statement because no work was developed with human samples. Strains were isolated directly from the patients to plates.<br>Strains were collected not only for this study, but also for diagnosing of infection.<br>Patient identifying information was collected by medical doctors as part of the routine hospital patient care procedure, and a number was assigned to each patient.<br>Information arrived to the laboratory with this number after isolating and identifying all strains.<br>Patient consents for collecting their clinical signs, medical histories, and characteristics were obtained during de admission of the hospital as a part of the routine hospital patient care procedure.

Powered by Editorial Manager® and ProduXion Manager® from Aries Systems Corporation

**Format for specific study types**<br><br>**Human Subject Research (involving human participants and/or tissue)**<br>• Give the name of the institutional review board or ethics committee that approved the study<br>• Include the approval number and/or a statement indicating approval of this research<br>• Indicate the form of consent obtained (written/oral) or the reason that consent was not obtained (e.g. the data were analyzed anonymously)<br><br>**Animal Research (involving vertebrate animals, embryos or tissues)**<br>• Provide the name of the Institutional Animal Care and Use Committee (IACUC) or other relevant ethics board that reviewed the study protocol, and indicate whether they approved this research or granted a formal waiver of ethical approval<br>• Include an approval number if one was obtained<br>• If the study involved *non-human primates*, add *additional details* about animal welfare and steps taken to ameliorate suffering<br>• If anesthesia, euthanasia, or any kind of animal sacrifice is part of the study, include briefly which substances and/or methods were applied<br><br>**Field Research**<br><br>Include the following details if this study involves the collection of plant, animal, or other materials from a natural setting:<br>• Field permit number<br>• Name of the institution or relevant body that granted permission	
**Data Availability**<br><br>Authors are required to make all data underlying the findings described fully available, without restriction, and from the time of publication. PLOS allows rare exceptions to address legal and ethical concerns. See the PLOS Data Policy and FAQ for detailed information.	Yes - all data are fully available without restriction

A Data Availability Statement describing where the data can be found is required at submission. Your answers to this question constitute the Data Availability Statement and **will be published in the article**, if accepted.<br><br>**Important:** Stating 'data available on request from the author' is not sufficient. If your data are only available upon request, select 'No' for the first question and explain your exceptional situation in the text box.<br><br>Do the authors confirm that all data underlying the findings described in their manuscript are fully available without restriction?	
**Describe where the data may be found in full sentences. If you are copying our sample text, replace any instances of XXX with the appropriate details.**<br><br>• If the data are **held or will be held in a public repository**, include URLs, accession numbers or DOIs. If this information will only be available after acceptance, indicate this by ticking the box below. For example: *All XXX files are available from the XXX database (accession number(s) XXX, XXX.*).<br>• If the data are all contained **within the manuscript and/or Supporting Information files**, enter the following: *All relevant data are within the manuscript and its Supporting Information files.*<br>• If neither of these applies but you are able to provide **details of access elsewhere**, with or without limitations, please do so. For example:<br><br>*Data cannot be shared publicly because of [XXX]. Data are available from the XXX Institutional Data Access / Ethics Committee (contact via XXX) for researchers who meet the criteria for access to confidential data.*<br><br>*The data underlying the results presented in the study are available from (include the name of the third party*	All relevant data are within the manuscript and its Supporting Information files.

and contact information or URL).<br>• This text is appropriate if the data are owned by a third party and authors do not have permission to share the data.<br><br>* typeset	
Additional data availability information:	

Manuscript Click here to access/download;Manuscript;manuscript.docx

Study of susceptibility to antibiotics and molecular characterisation of high virulence *Staphylococcus aureus* strains isolated from a rural hospital in Ethiopia

Cristina Verdú-Expósito[1*], Juan Romanyk[2], Juan Cuadros-González[2], Abraham TesfaMariam[3], José Luis Copa-Patiño[1], Jorge Pérez-Serrano[1], Juan Soliveri[1]

1. Department of Biomedicine and Biotechnology, University of Alcalá, 28805 Alcalá de Henares, Madrid, Spain.
2. Microbiology Service, Hospital Universitario Príncipe de Asturias, Alcalá-Meco, s/n, 28805 Alcalá de Henares, Madrid, Spain.
3. Department of General Medicine, Gambo General Rural Hospital, West-Arsi, Ethiopia.

* Corresponding author. Tel.: +34 606528592
E-mail address: cristina.verdu@uah.es

Abstract

We characterised 80 *Staphylococcus aureus* strains isolated from human patients with SSTIs at a rural hospital in Ethiopia.

Susceptibility to antibiotic of all strains was tested. The MLST method was used to type and a phylogenetic analysis was conducted employing the sequences of 7 housekeeping genes. PCR amplification was used to investigate the presence of the following virulence genes in all strains: *hla* (α-haemolysin), *tstH* (toxic shock syndrome toxin), *luk PV* (Panton-Valentine leukocidin), *fnbA* (fibronectin binding protein A) and *mecA* (methicillin resistance).

Most of the strains were resistant to penicillin and ampicillin, but only 3 strains were resistant to oxacillin, and 1 of them was a true MRSA. The MLST results showed a high diversity of sequence types (ST), 55% of which were new, and ST152 was the most prevalent. A phylogeny study showed that many of the new STs were phylogenetically related to other previously described STs, but bore little relationship to the only ST from Ethiopia described in the database. Virulence gene detection showed a high prevalence of strains encoding the *hla*, *fnbA* and *pvl* genes (98.77%, 96.3% and 72.84%,

respectively), a low prevalence of the *tst* gene (13.58%) and a markedly low prevalence of MRSA (1.25%).

S. aureus strains isolated from patients in a rural area in Ethiopia showed low levels of antibiotic resistance, except to penicillin. Moreover, this study reveals new STs in Eastern Africa that are phylogenetically related to other previously described STs, and confirm the high prevalence of the *pvl* gene and the low prevalence of MRSA on the continent.

Key words

Staphylococcus aureus – Ethiopia – MLST – resistance - Phylogeny – Virulence

Introduction

Staphylococcus aureus is a gram positive bacterium with carrier rates of 25-50% in the general population as a commensal microorganism, but it can also become an opportunistic pathogen under certain circumstances. Consequently, it is not only the cause of community-acquired infection (CAI), but is also one of the most important aetiological agents of hospital-acquired infection (HAI) (Steinberg et al., 2011). *S. aureus* presents high plasticity, conferring an exceptional capacity to incorporate genetic material from other strains, and acquire new infection characteristics related to antibiotic resistance and virulence (Ã et al., 2010). Hence, phylogenetic studies are very useful to determine the relationship and evolution of different strains, even from different parts of the world (Musser & Kapur, 1992).

As a result of its versatility and adaptability in the antibiotic era, *S. aureus* has acquired resistance to most of the antibiotics used to treat it (Stryjewski & Corey, 2014). The clearest example of this are the methicillin-resistant *Staphylococcus aureus* strains (MRSA), which express the chromosomal gene *mecA* that encodes a PBP2a transpeptidase, which shows a reduced affinity for all available beta-lactam agents, including penicillin (Filipe et al., 2001; Lim & Strynadka, 2002). This gene is located in a small mobile genomic element known as the staphylococcal cassette chromosome (SCC), and is thought to be acquired through horizontal transfer from coagulase-

negative staphylococci (Ito & Hiramatsu, 1998). MRSA is responsible for a large number of serious infections and hospital-related deaths in developed countries (Deleo, Otto, Kreiswirth, & Chambers, 2010; Kejela & Bacha, 2013), but are also starting to be detected in developing countries (Abdulgader, Shittu, Nicol, & Kaba, 2015; Eshetie et al., 2016).

S. aureus can also produce a variety of thermostable extracellular protein toxins which behave as virulence factors, including superantigens, haemolysins, leukocidins. The most important of them is the Panton-Valentine leukocidin (PVL), which is a cytotoxin that forms pores in the membrane, and has been associated with furuncles, cutaneous abscesses and severe necrotic skin infections, increasing disease severity (Couppie, Cribier, Prévost, Grosshans, & Piémont, 1994; Etienne, 2005). This toxin requires the assembly of two polypeptides, LukS-PV and LukF-PV, into a hetero-oligomeric pore (Labandeira-Rey et al., 2007). Although PVL has been related to both community-acquired (CA) MSSA and MRSA (CA-MSSA and CA-MRSA, respectively), an exceptionally large number of MSSAs which express PVL has been isolated in Africa; therefore, this continent has been considered an endemic region for PVL-positive *S. aureus* strains (Schaumburg et al., 2014).

As noted above, haemolysins are also an important virulence factor. For example, α-haemolysin (Hla) is a pore-forming cytotoxin that can lead to cell lysis and death, producing abscesses in different parts of the body (Hassel et al., 2015). This toxin is also closely associated with skin and soft tissue infections (SSTI), because it plays a major role in epithelial injury during *S. aureus* infection (Berube et al., 2014). It is encoded by the *hla* gene, which is located in the core genome (Tavares et al., 2014).

Another important toxin that affects strain virulence is the toxic shock syndrome toxin (TSST), which is encoded by the *tstH* gene(Plata, Rosato, & Wegrzyn, 2009; Zschöck, Botzler, Blöcher, Sommerhäuser, & Hamann, 2000). This exotoxin has multiple biological properties, including the capacity to induce fever, hypotension and lethal shock, (Takeuchi, Ishiguro, Ikegami, Kaidoh, & Hayakawa, 1998). The main cause of toxic shock syndrome in humans is the TSST-1 protein (toxic shock syndrome toxin 1) (Fueyo et al., 2005).

S. aureus pathogenesis is essentially related to the expression of surface fibronectin binding proteins (FnBPs). FnBP-A protein is widely found in body fluids, blood clots and extracellular matrices and its main function appears to be related to the capacity for protein-mediated adhesion of most eukaryotic cells, but it also binds *S. aureus* cells and serves as an adhesion substrate for various microorganisms. This represents a crucial step in the colonisation of host tissue and development of infection (Kuusela, 1978; Wadstrom et al., 1985; Woods et al., 1986). FnBP-A toxin is a glycoprotein formed by two similar subunits, and is encoded by the gene *fnbA* located in the chromosomal DNA (Peacock, Thomas, Foster, Day, & Berendt, 2000).

Although *S. aureus* infections represent a serious pathogen problem worldwide, studies have largely focused on affluent regions and developed countries, and little information is available on developing countries, especially in East Africa, and particularly in Ethiopia (Shibabaw, Abebe, & Mihret, 2014). The Gambo General Rural Hospital is located in the Oromia Region (Ethiopia). Between 2014 and 2018, 80 strains identified as *Staphylococcus aureus* were isolated from patients with extensive, deep subcutaneous purulent lesions, mostly community-acquired SSTIs, which caused recurrent infection in some cases. These strains were mostly methicillin-susceptible (CA-MSSA), except for one resistant CA-MRSA. It is not known if this high virulence was due to the effect of socio-economic and nutritional conditions of the indigenous population or to intrinsic or acquired virulence factors.

The general aim of the present study was to characterise the strains isolated from a rural hospital in Ethiopia in order to determine the origin of their virulence. To this end, susceptibility to antibiotic was tested, and strains were typified by MLST and their sequences were used to study their phylogenetic evolution. Then, the presence of several virulence genes was investigated to determine the origin of the strains' virulence.

Materials and Methods

Strains

Between June 2014 and June 2018, 80 *Staphylococcus aureus* strains were isolated at Gambo General Rural Hospital from different human samples, mainly SSTIs but also from some patients with osteomyelitis, leprosy and pyomiositis. In general, strains appeared to be highly virulent and all patients presented deep and extensive lesions, mostly ulcers, and recurrent infection in some cases.

Four different *S. aureus subsp. aureus* strains from the Spanish Type Culture Collection (CECT 976, CECT 957, CECT 435 and CECT 4439) were used as reference controls for different experiments.

In this study, the DNA of each strain was extracted using the UltraClean® Microbial DNA Isolation Kit (MoBio Laboratories Inc., 12224-250) according to the manufacturer's instructions, and this DNA was used for all analyses.

Clinical signs, medical histories and patient characteristics were collected to study any possible relationship with strain characteristics. This study was authorised by the Gambo General Rural Hospital management.

Identification of strains

First, the preliminary identification of the strains was confirmed by MALDI-TOF (matrix-assisted laser desorption/ionisation-time-of-flight) (MALDI Biotyper System, Bruker), and by partial amplification of 16S gene using the 27F and 1492R primers and sequencing, according to the procedure described by Lane (1991). All sequences were compared against each other for similarity using NCBI-BLAST and the nucleotide collection database (http://blast.ncbi.nlm.nih.gov/Blast.cgi).

Susceptibility to antibiotics

Susceptibility to antibiotics was analysed by the microdilution broth method using an automated MicroScan system and interpreted with EUCAST expert rules. Briefly, strains were inoculated on Trypticase^{TM} Soy Agar II with 5% Sheep Blood plates (Becton Dickinson, 254053) and incubated for 24 h at 37°C. An isolated colony of each strain was selected for inoculation on a MicroScan®-Pos MIC Panel Type 33 (Beckman Coulter,

B1016-173), a multicell panel with different antibiotics and concentrations. Panels were incubated for 20 h at 37°C in the MicroScan autoScan4 system (Siemens, B1018-280) with the WalkAway-96 system, which measured final absorbance at 450 nm in each well to determine the presence or absence of growth and therefore susceptibility to antibiotics. The antibiotics tested were as follows: penicillin, ampicillin, amoxicillin + clavulanic acid, oxacillin, cefoxitin, ceftaroline, imipenem, gentamicin, tobramycin, amikacin, vancomycin, teicoplanin, daptomycin, ciprofloxacin, levofloxacin, moxifloxacin, erythromycin, clindamycin, Synercid, pristinamycin, tetracycline, minocycline, linezolid, trimethoprim-sulphamethoxazole, rifampicin, fosfomycin, mupirocin and chloramphenicol.

Moreover, age of patients was taking into account for comparing with the prevalence of resistances found in each strain.

Strain typing by MLST

MLST analysis was used to typify all strains (Jolley, Bray, & Maiden, 2018). For that purpose, amplification of 7 housekeeping genes (*arc*, *aro*, *glp*, *gmk*, *pta*, *tpi* and *yqi*) was carried out using the PCR conditions described on the *S. aureus* MLST website, and sequences were obtained by the Sanger method using multi-capillary sequencing (ABI PRISM 3130XL, Applied Biosystems). Then, sequences for each gene from each strain were analysed with BioEdit software and compared with the *S. aureus* MLST database (https://pubmlst.org/saureus/). In accordance with this database, one allele was assigned to each sequence, and the combination of 7 alleles comprised a specific sequence type (ST) for each strain.

Phylogeny study

Firstly, phylogenetic analysis with all the strains and ST that belong to *S. argenteus* and *S. schweitzeri* was performed to discard that strains belong to these species rather than *S. aureus*.

Then, another phylogenetic analysis was realized with each ST determined previously by MLST typing to know their phylogenetic relations.

Both phylogenetic studies were conducted using the sequences obtained from the 7 housekeeping genes employed in the MLST study. First, the 7 sequences were concatenated in order to create a unique sequence for each strain, and then a multiple alignment of all these sequences was performed with the BioEdit software. G-block software (version 0.91b) was used to eliminate highly variable areas of the sequences. Next, the appropriate substitution model was selected using the jModelTest application (version 2.1.10). Lastly, a maximum likelihood tree was generated and analysed using Mega X software.

Virulence genes detection by PCR

The virulence genes analysed (and the products they codify for) were *hla* (α-haemolysin), *tstH* (toxic shock syndrome toxin), *lukS/F-PV* (S and F components of Panton-Valentine leukocidin), *fnbA* (fibronectin binding protein A) and *mecA* (methicillin resistance). The analyses were carried out by partial PCR amplification of the genes in all strains, under the conditions described in annex I (Delgado et al., 2011). To detect amplified genes, PCR products were resolved using electrophoresis in 1.2% agarose gel (D1 Low EEO, Conda, 8018). The reference controls used to detect each virulence gene were: CECT 976 for *hla*, CECT 957 for *tst*, CECT 435 for *lukS/F-PV*, CECT 976 for *fnbA* and CECT 4439 for *mecA*, all from the Spanish Type Culture Collection (Spanish initials: CECT). In addition, a molecular weight standard (ϕX174-Hae III digest, Takara Bio Inc., 3405A) was included to determine the size of the amplified fragments.

A relationship between MLST STs and the presence of virulence genes was investigated using Canoco software (version 5.12), conducting a constrained redundancy analysis (RDA).

Results

Strain identification

Besides MALDI-TOF identification of *Staphylococcus aureus*, the 16S ribosomal sequences obtained from each sample were compared in the database and

identification of the samples as *S. aureus* was confirmed with at least 98% identity for all of them.

Susceptibility to antibiotics

Results for the MicroScan analysis of all strains are shown in Table 1. Resistance to β-lactam antibiotics was: 88.75% to penicillin, 86.25% to ampicillin, 3.75% to oxacillin and 1.25% to cefoxitin and imipenem. No resistance was detected to ceftaroline.

With regard to β-lactam antibiotics, 3 of the total strains showed resistance to oxacillin, but only 1 of them (strain 73) showed resistance to cefoxitin and imipenem, but also to quinolones (ciprofloxacin, levofloxacin and moxifloxacin).

It is important to note that strain 73 showed the most resistance to the antibiotics used in the assay (13 of 29), and was the only strain isolated that showed resistance to cefoxitin, imipenem, trimethoprim-sulphamethoxazole and to all the MLS group (macrolides, lincosamides and streptogramines).

Finally, strains that belonged to adult patients presented more number of resistances than children patients.

Strain typing by MLST

The results of MLST typing indicated high diversity, and according to the database, over half of the strains (N=44, 55%) presented new STs (N). Most of the new STs (38) presented new allele combinations that did not correspond to any ST in the database, while for the remaining 6 STs, there was no match in the MLST database for at least one of the 7 sequences studied and they could not be assigned to any described allele. All these new STs were submitted to database, where a new number of ST was assigned for each one. Most of the new STs (34) comprised only 1 strain, but 4 of them grouped various strains: ST N04 (strains 28, 29, 65 and 83), ST N34 (strains 56, 59 and 74), ST N33 (strains 39 and 46) and ST37 (strains 79 and 84).

We also defined a number of new alleles described for each gene: 2 for the *arc*, *aro* and *yqi* genes, 1 for *glp* and *pta*, and 0 for *gmk* and *tpi*.

Among the STs previously defined in the database (45%), 10 only included 1 strain, another two comprised 2 strains (ST5 and ST3224) and ST121 and ST15 comprised 3 and 4 strains, respectively. The sequence type ST152 was the most prevalent (15 strains) in this study, although with a low percentage (18.75%) with respect to all the strains isolated (Table 2).

Phylogeny study

A phylogenetic study was conducted using concatenated sequences of MLST housekeeping genes for each strain.

First phylogenetic study confirmed that none of the tested strains belonged to *S. argenteus* or *S. schweitzeri*.

About the second phylogenetic study, after aligning all sequences and discarding all hypervariable regions, an analysis was performed with jModelTest software, selecting the general time-reversible model (GTR) and including invariable sites (+I) and rate of variation across sites (+G) as the best substitution model. This model was used to generate a maximum likelihood (ML) tree with Mega software.

In the ML tree (Figure 1), 15 clusters could be differentiated. The largest group comprised 11 ST, six of them were new, but very similar to other already described. One of these groups (ST N30, N31 and N32) appeared external to the others with a different but closely related origin of the tree. ST N8 also appeared in an individual branch, but with a common branch point to the rest of the strains. The reference strain from Ethiopia (ST727) appeared in a group with ST N10, and this group was clearly distant from the rest, with a common branch except for the most external group mentioned previously.

Virulence gene detection

The results of PCR virulence detection are shown in Table 4. The most prevalent gene was *hla*, which was detected in all strains except number 24 (thus, 98.77% were α-haemolysin positive strains). In contrast, strain number 73 was the only one that presented the *mecA* gene (1.25% of strains) and one of the three samples did not present the *fnbA* gene (together with strains 15 and 72), indicating that 96.3% of strains

were positive for fibronectin binding protein A. In addition, a high percentage of strains (72.84%, 59 strains) showed the *pvl* gene. In contrast, the *tst* gene showed a low prevalence with only 11 strains (13.58%).

A correlational analysis using Canoco software indicated that in general there was no relationship between the presence of virulence genes and MLST STs, with an explained variance of 66.73% with 2 axes, especially in new STs, where virulent genes were highly dispersed (data not shown).

Discussion

In contrast to the rest of the world, the picture of the spread of *Staphylococcus aureus* infections in Africa is unclear, especially in Ethiopia, where the few studies available were performed in densely populated urban areas (Eshetie et al., 2016; Schaumburg, Alabi, Kaba, et al., 2014).

The study reported here is the first staphylococcal epidemiology study to be conducted in a rural region in Ethiopia, with low population density. Between 2014 and 2018, in Gambo General Rural Hospital, we isolated 80 *S. aureus* strains from clinical samples. All of them caused highly virulent infections with deep and extended lesions, and recurrent infections in some cases.

One of the most important virulence factors in *S. aureus* is the presence of antibiotic resistance. Penicillin was the first and most effective antibiotic against *S. aureus* infections, but also the first resistance emerged (Massova & Mobashery, 1998). In our study, most of the strains (88%) showed resistance to penicillin and ampicillin, a similar percentage to that found in other studies conducted in Ethiopia (Dilnessa & Bitew, 2016). Moreover, 3 of the total isolated strains (38, 43 and 73) exhibited resistance to oxacillin and 1 of them (strain 73) was also resistant to imipenem and cefoxitin. This result suggested that strain 73 was a true MRSA, and this fact was confirmed by amplification and detection of *mecA* gene by PCR, during virulence genes detection procedure, which confers resistance to methicillin (MRSA) and to oxacillin, imipenem and cefoxitin, the latter being a good surrogate marker for MRSA detection. In addition, MRSA strain 73 was the only strain resistant to quinolones. It has been suggested that

fluoroquinolones themselves may predispose patients to infection with MRSA (Wagenlehner et al., 2011). The percentage of MRSA and resistances to quinolones (ciprofloxacin, levofloxacin or moxifloxacin) obtained in our study was notably lower (1.25%) than that reported in other studies conducted in Ethiopia (Abera, Alem, & Beyene, 2008). This difference may be due to the fact that our study was carried out in a rural area with predominantly community-acquired infections, whereas the other two studies were conducted in a crowded hospital using samples mainly collected from surgical wounds, suggesting hospital-acquired MRSA strains.

On other hand, there may be several explanations for the phenotype of the strains (38 and 43) resistant to penicillin, ampicillin, amoxicillin + clavulanic acid and oxacillin, but susceptible to cefoxitin and imipenem, but further studies will be required to elucidate the precise mechanism of resistance (Tadesse et al., 2017).

Moreover, the collected strains were tested against ceftaroline. All of them were susceptible, probably because it is a fifth-generation cephalosporin that is resistant to *S. aureus* β-lactamase and has a high affinity for PBPs, including the PBP2a present in MRSA (Horcajada & Cantón, 2014).

The total percentage of aminoglycoside-resistant strains detected (15 - 18%) was similar to that found in other studies conducted in Africa (Tadesse et al., 2017). Resistance to aminoglycosides (gentamicin, tobramycin and amikacin) could be explained by various enzymes which selectively modify their structure and give resistance to one or more aminoglycosides. For example, the enzymes acetyltransferase and 2''-O-phosphotransferase AAC(6')-APH(2'') were probably the most common in the aminoglycoside-resistant strains in our study, because 80% of them were resistant to the three antibiotics mentioned above (Schmitz et al., 1999).

The results for the presence of tetracycline resistance showed that 52.5% of the tested strains were resistant solely to tetracycline and 6.25% to tetracycline and minocycline. Tetracycline resistance could be due to active efflux, while the 6.25% resistant to both tetracycline and minocycline could be explained by ribosomal protection (Grossman, 2016). These results are similar to those described as average in the African continent (Tadesse et al., 2017).

The analysis of resistance to the MLS_b group (macrolides, lincosamides and streptogramine$_b$) determined that 28.75% of strains were only resistant to the lincosamide clindamycin, perhaps due to the presence of the *lnu*(A) gene, which is very similar that results found in other studies conducted in South Africa (30%) (Lozano et al., 2012). However, resistance to both clindamycin and erythromycin (18%) could be explained by the presence of genes *erm*, which includes resistance to macrolides, lincosamides and streptogramin$_b$ (Li, Feng, Zhang, Xue, & Zhao, 2015). All strains were susceptible to Sinercyd, a combination of streptogramins A and B, the first of which is not affected by *erm* ribosomal modifications.

In contrast to the high prevalence of trimethoprim-sulphamethoxazole resistance reported in the literature (43 - 84%), we obtained a low percentage of resistant strains (1.25%) (Tadesse et al., 2017). However, research on *S. aureus* imported from Africa strongly suggests that trimethoprim-sulphamethoxazole resistance is emerging around the globe (Nurjadi et al., 2015).

The use of antibiotics in a rural area in Ethiopia is more restricted than in the country's large cities and economic centres, and under these conditions, we would expect to find lower levels antibiotic resistance. Our results confirmed this hypothesis and an analysis of the data showed that resistances were more common in adult patients, who would have been treated more frequently than children with antibiotics. This finding has also been described in urban areas and in developed countries, and in our case was especially notable in strain 73, the most multiple-resistant strain, which was obtained from an adult leprosy patient who had previously been treated repeatedly with different antibiotics.

Sequencing genomes from different *S. aureus* strains revealed that the diversity of genes was high, with 22% belonging to variable regions, rendering the pathogen highly versatile (Lindsay & Holden, 2004). Hence, surveillance of all *S. aureus* infections around the world is very important to improve our knowledge of their virulence, predict the evolution of strains and infection characteristics and prevent the global spread of multi-resistant or harmful *S. aureus*.

One important tool for surveillance studies is MLST, which uses an online database not only for identification but also to provide knowledge about molecular epidemiology and the global spread of virulent or antibiotic resistant isolates of bacterial pathogens (Enright & Spratt, 1999). This analysis has become the method of choice for molecular typing of many bacteria, including *S. aureus*, and for comparing strains around the world (Feil et al., 2003; Leeuwen et al., 2003).

We found a high diversity of STs in general and 44 strains with new STs, suggesting that they did not belong to the same outbreak. This wide diversity has been reported previously in a review of MLST studies conducted in Africa which mainly focused on Central and West Africa. The review identified ST30, ST121 and 2ST15 as the most prevalent *S. aureus* isolates, all of which were MSSA (Schaumburg et al., 2014). We found two of these (ST121 and ST152) in our study, and ST152 was the most prevalent of the two, with 15 strains (18.75%). The MLST database describes 31 isolates of ST152, most of them from Central-West and North Africa, but none of them from Ethiopia. It is noteworthy that the strains identified as ST152 were collected in different years, which could confirm the stability of this ST in this region.

In our study, ST15 and ST121 were also found with 4 and 3 strains, respectively. The MLST database describes 486 isolates of ST15 and 104 isolates of ST121, and both have been found in different parts of the world, including Africa, mainly in the Western region.

Some of the new STs were similar to other, previously described allelic profiles but contained 1 or 2 different alleles: 9 of them were similar to ST121, another 7 to ST5, 6 to ST15 and only 1 to ST152.

The only ST in Ethiopia described in the MLST database is ST727. Hence, ours is the first study in the Eastern region of Africa to identify not only new STs but also STs previously described in other regions of Africa. Moreover, the count of new alleles found in each of the 7 genes showed that the most variable gene in our strains was probably the last one (*yqi*), while *gmk* and *tpi* seemed to be more conserved genes.

Despite the high diversity observed with MLST analysis, phylogenetic studies showing that most of the strains were grouped into clusters and that many of the new STs were

phylogenetically related to other, previously described STs. However, a comparison with the only ST described in Ethiopia according to the MLST database demonstrated that our strains were clearly dissimilar (except ST N10) and presented a very different evolution.

The virulence analysis was focused on detecting the main virulence genes of *S. aureus*: *hla*, *tstH*, *lukS/F-PV*, *fnbA* and *mecA*. This analysis indicated a high percentage (72.84%) of PVL-positive strains. One explanation for this high percentage might be that the clinical symptoms were mainly cutaneous (SSTI), because PVL is usually associated with furuncles, cutaneous abscesses and severe necrotic skin infections (Couppie et al., 1994; Cribier et al., 1992; Gauduchon et al., 1999). However, this high percentage of PVL-positive strains is in agreement with previous studies conducted in other African countries (usually on MSSA), suggesting that there is a high prevalence of *S. aureus* strains presenting genes encoding PVL on this continent (Breurec et al., 2010; Egyir et al., 2014; Ruimy et al., 2008). It is considered an endemicity that could become a reservoir for the emergence of PVL-positive strains. Moreover, it is important to note that in our study, we found a relationship between ST152 and the presence of PVL. This relationship was described in the first studies with a CA-MRSA as a sporadic PVL producer, mostly located in Central Europe. However, later studies in Nigeria and Mali with PVL-positive MSSA ST152 isolates have suggested that ST152 divergence might be the result of a MSSA clone originating in Africa that migrated to Central Europe and acquired antibiotic resistance there, similar to MRSA (Okon et al., 2009; Ruimy et al., 2008). This could explain the prevalence of these infections in travellers from Africa, and the strong relationship between ST152 and PVL producers (Rasigade et al., 2014; Zanger et al., 2012).

The *hla* gene was detected in all strains except one (24). The high prevalence (98.75%) of this gene was similar to that found in other studies on *S. aureus* infections in Africa (Amissah et al., 2017; Kateete et al., 2011) and throughout the world (Tabor et al., 2016). It is also important to note that strain 24 was also the most phylogenetically differentiated from the rest.

Only three of the studied strains did not show the *fnba* gene (15, 24 and 72). This is a high percentage (96.3%) of fnba-encoding genes, but is in agreement with previous studies conducted in other African countries (Oosthuysen, Orth, Lombard, Sinha, &

Wasserman, 2014; Vubil et al., 2017). The high prevalence of α-haemolysin and fibronectin binding protein A is not unusual and could explain the high virulence of these infections, as they play a crucial role in colonisation and infection (Hassel et al., 2015; Woods et al., 1986).

We found a low prevalence (13.58%) of strains encoding the *tst* gene, which is similar to that found in another study in Congo (17.5%) (De Boeck et al., 2015) and to the prevalence of carriers and clinical isolates in Europe (15–25%) (Becker et al., 2003; Megevand et al., 2010). Although TSST-positive infections were initially associated with menstruation, the origin of staphylococcal TSS is diverse, and the most common foci of infection in non-menstrual cases in developed countries are SSTIs (Devries et al., 2011; Hajjeh et al., 1999). Moreover, it is important to emphasise that most of the samples (9 out of 11; 82%) were collected in the same year (2014), suggesting that there was a high rate of acquisition of this gene during that period.

Statistical analysis showed that there was no relationship between the presence of virulence genes and MLST STs. This could be explained by differences in the acquisition of virulence genes described previously and variability in MLST sequences, but might also be due to the high diversity of STs found.

To sum up, the present study shows a high diversity of *S. aureus* strains in the same rural region, which enhances our knowledge of this kind of infection in Eastern Africa.

Conclusions

- This is one of the first studies on *Staphylococcus aureus* epidemiology in a rural region in Ethiopia.
- The prevalence of resistance, especially of MRSA, was low compared with that reported in studies conducted in other parts of Ethiopia and Africa.
- MLST analysis showed high diversity of STs and 44 strains with new STs, most of which (new and previously defined STs) are described for the first time in Ethiopia.
- Phylogeny studies determined phylogenetic relationships between the new STs and the previously described STs, but far from the only Ethiopian ST in the database.

- Virulence gene detection showed a high prevalence of strains encoding *pvl*, *hla* and *fnba*, a low prevalence of the *tss* gene, and a markedly low prevalence of MRSA.
- We found no relationship between STs and the presence of virulence genes, but did observe a relationship between ST152 and the PVL gene, highly defined in other parts of Africa.

Bibliography

This publication made use of the *Staphylococcus aureus* MLST website (https://pubmlst.org/ saureus/) sited at the University of Oxford (Jolley et al. Wellcome Open Res 2018, 3:124).

Ã, A. Z., Kusch, H., Degner, M., Jaglitz, S., Sibbald, M. J. J. B., Arends, J. P., … Engelmann, S. (2010). Proteomics uncovers extreme heterogeneity in the *Staphylococcus aureus* exoproteome due to genomic plasticity and variant gene regulation. *Proeomics*, *10*, 1634–1644. https://doi.org/10.1002/pmic.200900313

Abdulgader, S. M., Shittu, A. O., Nicol, M. P., & Kaba, M. (2015). Molecular epidemiology of Methicillin-resistant *Staphylococcus aureus* in Africa: A systematic review. *Frontiers in Microbiology*, *6*(APR). https://doi.org/10.3389/fmicb.2015.00348

Abera, B., Alem, A., & Beyene, B. B. (2008). Methicillin-resistant strains of *Staphylococcus aureus* and coagulase- negative staphylococus from clinical isolates at Felege Hiwot Refferal Hospital, North West Ethiopia. *Ethiopian Medical Journal*, *46*(1), 149–154.

Amissah, N. A., Chlebowicz, M. A., Ablordey, A., Tetteh, C. S., Prah, I., Werf, T. S. van der, … Rossen, J. W. (2017). Virulence potential of *Staphylococcus aureus* isolates from Buruli ulcer patients. *International Journal of Medical Microbiology*, *307*, 223–232. https://doi.org/10.1016/j.ijmm.2017.04.002

Becker, K., Friedrich, A. W., Lubritz, G., Weilert, M., Peters, G., & von Eiff, C. (2003). Prevalence of genes encoding pyrogenic toxin superantigens and exfoliative toxins among strains of *Staphylococcus aureus* isolated from blood and nasal specimens. *Journal of Clinical Investigation*, *41*(4), 1434–1439. https://doi.org/10.1128/JCM.41.4.1434

Berube, B. J., Sampedro, G. R., Otto, M., & Wardenburg, J. B. (2014). The psmα locus regulates production of *Staphylococcus aureus* alpha-toxin during infection. *Infection and Immunity*, *82*(8), 3350–3358. https://doi.org/10.1128/IAI.00089-14

Breurec, S., Fall, C., Pouillot, R., Boisier, P., Brisse, S., Djibo, S., … Fonkoua, M. C. (2010). Epidemiology of methicillin-susceptible *Staphylococcus aureus* lineages in five major African towns : high prevalence of Panton – Valentine leukocidin genes. *Clinical Microbiology and Infection*, *17*, 633–639.

Couppie, P., Cribier, B., Prévost, G., Grosshans, E., & Piémont, Y. (1994). Leukocidin

from *Staphylococcus aureus* and cutaneous infections: an epidemiologic study. *Arch Dermatol*, *130*, 1208–1209.

Cribier, B., Prevost, G., Couppie, P., Finck-Barbançon, V., Grosshans, E., & Piemont, Y. (1992). *Staphylococcus aureus* Leukodicin: a new virulence factor in cutaneous infections? *Dermatology*, *185*, 175–180.

De Boeck, H., Vandendriessche, S., Hallin, M., Batoko, B., Alworonga, J. P., Mapendo, B., … Jacobs, J. (2015). *Staphylococcus aureus* nasal carriage among healthcare workers in Kisangani , the Democratic Republic of the Congo. *European Journal of Clinical Microbiology Infectious Diseases*, *34*, 1567–1572. https://doi.org/10.1007/s10096-015-2387-9

Deleo, F. R., Otto, M., Kreiswirth, B. N., & Chambers, H. F. (2010). Community-associated methicillin-resistant *Staphylococcus aureus*. *Lancet*, *375*(9725), 1557–1568. https://doi.org/10.1016/S0140-6736(09)61999-1.Community-associated

Delgado, S., García, P., Fernández, L., Jiménez, E., Rodríguez-Baños, M., del Campo, R., & Rodríguez, J. M. (2011). Characterization of *Staphylococcus aureus* strains involved in human and bovine mastitis. *FEMS Immunology and Medical Microbiology*, *62*(2), 225–235. https://doi.org/10.1111/j.1574-695X.2011.00806.x

Devries, A. S., Lesher, L., Schlievert, P. M., Rogers, T., Villaume, L. G., Danila, R., & Lynfield, R. (2011). Staphylococcal toxic shock syndrome 2000 – 2006: epidemiology, clinical features, and molecular characteristics. *PLoS Medicine*, *6*(8). https://doi.org/10.1371/journal.pone.0022997

Dilnessa, T., & Bitew, A. (2016). Prevalence and antimicrobial susceptibility pattern of methicillin resistant *Staphylococcus aureus* isolated from clinical samples at Yekatit 12 Hospital Medical College, Addis Ababa, Ethiopia. *BMC Infectious Diseases*, *16*(398), 1–9. https://doi.org/10.1186/s12879-016-1742-5

Dobrindt, U. (2002). Whole genome plasticity in pathogenic bacteria. *Current Opinion in Microbiology*, *4*(5), 550–557. https://doi.org/10.1016/s1369-5274(00)00250-2

Egyir, B., Guardabassi, L., Sørum, M., Nielsen, S. S., Kolekang, A., Frimpong, E., … Larsen, A. R. (2014). Molecular epidemiology and antimicrobial susceptibility of clinical *Staphylococcus aureus* from healthcare institutions in Ghana. *Plos One*, *9*(2), 3–9. https://doi.org/10.1371/journal.pone.0089716

Enright, M. C., & Spratt, B. G. (1999). Multilocus sequence typing. *Trends in Microbiology*, *7*(12), 482–487.

Eshetie, S., Tarekegn, F., Moges, F., Amsalu, A., Birhan, W., & Huruy, K. (2016). Methicillin resistant *Staphylococcus aureus* in Ethiopia: A meta-analysis. *BMC Infectious Diseases*, *16*(1). https://doi.org/10.1186/s12879-016-2014-0

Etienne, J. (2005). Panton-Valentine Leukocidin: a marker of severity for *Staphylococcus aureus* infection? *Clinical Infectious Diseases*, *41*(5), 591–593. https://doi.org/10.1086/432481

Feil, E. J., Cooper, J. E., Grundmann, H., Robinson, D. A., Enright, M. C., Berendt, T., … Day, N. P. J. (2003). How clonal is *Staphylococcus aureus*? *Journal of Bacteriology*,

185(11), 3307–3316. https://doi.org/10.1128/JB.185.11.3307

Filipe, R. R., Pinho, M. G., & Tomasz, A. (2001). Complementation of the essential peptidoglycan transpeptidase function of penicillin-binding protein 2 (PBP2) by the drug resistance protein PBP2A in *Staphylococcus aureus*. *Journal of Bacteriology*, *183*(22), 6525–6531. https://doi.org/10.1128/JB.183.22.6525

Fitzgerald, J. R., Sturdevant, D. E., Mackie, S. M., Gill, S. R., & Musser, J. M. (2001). Evolutionary genomics of *Staphylococcus aureus*: Insights into the origin of methicillin-resistant strains and the toxic shock syndrome epidemic. *Proceedings of the National Academy of Sciences of the United States of America (PNAS)*, *98*(15), 8821–8826.

Fueyo, J. M., Mendoza, M. C., & Martín, M. C. (2005). Enterotoxins and toxic shock syndrome toxin in *Staphylococcus aureus* recovered from human nasal carriers and manually handled foods: Epidemiological and genetic findings. *Microbes and Infection*, *7*(2), 187–194. https://doi.org/10.1016/j.micinf.2004.10.009

Gauduchon, V., Bes, M., Vandenesch, F., Piemont, Y., Lina, G., Etienne, J., … Godail-Gamot, F. (1999). Involvement of Panton-Valentine Leukocidin-producing *Staphylococcus aureus* in primary skin infections and pneumonia. *Clinical Infectious Diseases*, *29*(5), 1128–1132. https://doi.org/10.1086/313461

Grossman, T. H. (2016). Tetracycline antibiotics and resistance. *Cold Spring Harbor Perspectives in Medicine*, *6*. https://doi.org/10.1515/hsz-2013-0292

Hajjeh, R. A., Reingold, A., Weil, A., Shutt, K., Schuchat, A., & Perkins, B. A. (1999). Toxic Shock Syndrome in the United States: Surveillance Update, 1979-1966. *Emerging Infectious Diseases*, *5*(6), 807–810.

Hassel, B., Mæhlen, J., Dahlberg, D., Antal, E.-A., Goverud, I. L., Mariussen, E., & Tønjum, T. (2015). Staphylococcal α-hemolysin is neurotoxic and causes lysis of brain cells in vivo and in vitro. *NeuroToxicology*, *48*, 61–67. https://doi.org/10.1016/j.neuro.2015.03.001

Horcajada, J. P., & Cantón, R. (2014). Ceftarolina, un nuevo antimicrobiano de amplio espectro en la era de las multirresistencias. *Enfermedades Infecciosas y Microbiología Clínica*, *32*(2), 1–17.

Ito, T., & Hiramatsu, K. (1998). Acquisiton of methicillin resistance and progression of multiantibiotic resistance in Methicillin-Resistant *Staphylococcus aureus*. *Yonsei Medical Journal*, *39*(6), 526–533.

Jolley, K. A., Bray, J. E., & Maiden, M. C. J. (2018). Open-access bacterial population genomics: BIGSdb software, the PubMLST.org website and their applications [version 1; referees: 2 approved]. *Wellcome Open Research*, *3*(124), 1–20. https://doi.org/10.12688/wellcomeopenres.14826.1

Kateete, D. P., Namazzi, S., Okee, M., Okeng, A., Baluku, H., Musisi, N. L., … Najjuka, F. C. (2011). High prevalence of methicillin resistant *Staphylococcus aureus* in the surgical units of Mulago hospital in Kampala , Uganda. *BMC Research Notes*, *4*(326).

Kejela, T., & Bacha, K. (2013). Prevalence and antibiotic susceptibility pattern of methicillin-resistant *Staphylococcus aureus* (MRSA) among primary school children and prisoners in Jimma Town, Southwest Ethiopia. *Annals of Clinical Microbiology and Antimicrobials*, *12*(1), 1. https://doi.org/10.1186/1476-0711-12-11

Kuusela, P. (1978). Fibronectin binds to *Staphylococcus aureus*. *Nature*, *276*(December), 718–720.

Labandeira-Rey, M., Couzon, F., Boisset, S., Brown, E. L., Bes, M., Benito, Y., … Bowden, M. G. (2007). *Staphylococcus aureus* Panton-Valentine Leukocidin causes necrotizing pneumonia. In *Science* (Vol. 315).

Leeuwen, W. B. Van, Jay, C., Snijders, S., Durin, N., Lacroix, B., Verbrugh, H. A., … Belkum, A. Van. (2003). Multilocus Sequence Typing of *Staphylococcus aureus* with DNA Array Technology. *Journal of Clinical Microbiology*, *41*(7), 3323–3326. https://doi.org/10.1128/JCM.41.7.3323

Li, L., Feng, W., Zhang, Z., Xue, H., & Zhao, X. (2015). Macrolide-lincosamide-streptogramin resistance phenotypes and genotypes of coagulase-positive *Staphylococcus aureus* and coagulase-negative staphylococcal isolates from bovine mastitis. *BMC Veterinary Research*, *11*(168), 1–8. https://doi.org/10.1186/s12917-015-0492-8

Lim, D., & Strynadka, N. C. J. (2002). Structural basis for the β lactam resistance of PBP2a from methicillin-resistant *Staphylococcus aureus*. *Nature Structural Biology*, *9*(11). https://doi.org/10.1038/nsb858

Lindsay, J. A., & Holden, M. T. G. (2004). *Staphylococcus aureus*: superbug , super genome? *Trends in Microbiology*, *12*(8), 378–385. https://doi.org/10.1016/j.tim.2004.06.004

Lozano, C., Aspiroz, C., Sáenz, Y., Ruiz-García, M., Royo-García, G., Gómez-Sanz, E., … Torres, C. (2012). Genetic environment and location of the lnu(A) and lnu(B) genes in methicillin-resistant *Staphylococcus aureus* and other staphylococci of animal and human origin. *Journal of Antimicrobial Chemotherapy*, *67*(12), 2804–2808. https://doi.org/10.1093/jac/dks320

Mark C. Enright, Nicholas P. J. Day, Catrin E. Davies, Sharon J. Peacock, B. G. S. (2000). Multilocus Sequence Typing for characterization of Methicillin-Resistant and Methicillin-Susceptible clones of *Staphylococcus aureus*. *American Society for Microbiology*, *38*(3), 1008–1015. https://doi.org/10.1128/AAC.49.5.2098

Massova, I., & Mobashery, S. (1998). Kinship and diversification of bacterial penicillin-binding proteins and β-lactamases. *Antimicrobial Agents and Chemotherapy*, *42*(1), 1–17.

Megevand, C., Gervaix, A., Heininger, U., Berger, C., Aebi, C., Vaudaux, B., … Hitzler, M. (2010). Molecular epidemiology of the nasal colonization by methicillin-susceptible *Staphylococcus aureus* in Swiss children. *Clinical Microbiology and Infection*, *16*, 1414–1420. https://doi.org/10.1111/j.1469-0691.2009.03090.x

Musser, J. M., & Kapur, V. (1992). Clonal analysis of methicillin-resistant *Staphylococcus aureus* strains from intercontinental sources: association of the mec gene with divergent phylogenetic lineages implies dissemination by horizontal transfer and recombination. *Journal of Clinical Microbiology*, *30*(8), 2058–2063. Retrieved from NS -

Nurjadi, D., Schäfer, J., Friedrich-Jänicke, B., Mueller, A., Neumayr, A., Calvo-Cano, A., … Zanger, P. (2015). Predominance of dfrG as determinant of trimethoprim resistance in imported *Staphylococcus aureus*. *Clinical Microbiology and Infection*, *21*(12). https://doi.org/10.1016/j.cmi.2015.08.021

Oosthuysen, W. F., Orth, H., Lombard, C., Sinha, B., & Wasserman, E. (2014). In vitro characterization of representative clinical South African *Staphylococcus aureus* isolates from various clonal lineages. *New Microbes and New Infections*, *2*, 115–122.

Peacock, S. J., Thomas, M. G., Foster, T. J., Day, N. P. J., & Berendt, A. R. (2000). Clinical isolates of *Staphylococcus aureus* exhibit diversity in fnb genes and adhesion to human fibronectin. *Journal of Infection*, *41*(1), 23–31. https://doi.org/10.1053/jinf.2000.0657

Plata, K., Rosato, A. E., & Wegrzyn, G. (2009). *Staphylococcus aureus* as an infectious agent: Overview of biochemistry and molecular genetics of its pathogenicity. *Acta Biochimica Polonica*, *56*(4), 597–612. https://doi.org/20091925 [pii]

Rasigade, J.-P., Dumitrescu, O., & Lina, G. (2014). New epidemiology of *Staphylococcus aureus* infections in the Middle East. *Clinical Microbiology and Infection*, *20*(7), 587–588. https://doi.org/10.1111/1469-0691.12691

Ruimy, R., Maiga, A., Armand-lefevre, L., Maiga, I., Diallo, A., Ouattara, K., … Feil, E. J. (2008). The carriage population of *Staphylococcus aureus* from Mali is composed of a combination of pandemic clones and the divergent Panton-Valentine Leukocidin-positive. *Journal of Bacteriology*, *190*(11), 3962–3968. https://doi.org/10.1128/JB.01947-07

Schaumburg, F., Alabi, A. S., Kaba, H., Zoleko, R. M., Diop, D. A., Mackanga, J., … Becker, K. (2014). Transmission of *Staphylococcus aureus* between mothers and infants in an African setting. *Clinical Microbiology and Infection*, *20*, O390–O396. https://doi.org/10.1111/1469-0691.12417

Schaumburg, F., Alabi, A. S., Peters, G., & Becker, K. (2014). New epidemiology of *Staphylococcus aureus* infection in Africa. *Clinical Microbiology and Infection*, *20*(7), 589–596. https://doi.org/10.1111/1469-0691.12690

Schmitz, F. J., Fluit, A. C., Gondolf, M., Beyrau, R., Lindenlauf, E., Verhoef, J., … Jones, M. E. (1999). The prevalence of aminoglycoside resistance and corresponding resistance genes in clinical isolates of staphylococci from 19 European hospitals. *Journal of Antimicrobial Chemotherapy*, *43*(2), 253–259. https://doi.org/10.1093/jac/43.2.253

Shibabaw, A., Abebe, T., & Mihret, A. (2014). Antimicrobial susceptibility pattern of nasal *Staphylococcus aureus* among Dessie Referral Hospital health care workers,

Dessie, Northeast Ethiopia. *International Journal of Infectious Diseases*, *25*, 22–25. https://doi.org/10.1016/j.ijid.2014.03.1386

Steinberg, J. P., Clark, C. C., & Hackman, B. O. (2011). Nosocomial and Community-Acquired *Staphylococcus aureus* bacteremias from 1980 to 1993: impact of intravascular devices and methicillin resistance. *Clinical Infectious Diseases*, *23*(2), 255–259. https://doi.org/10.1093/clinids/23.2.255

Stryjewski, M. E., & Corey, G. R. (2014). Methicillin-resistant *Staphylococcus aureus*: An evolving pathogen. *Clinical Infectious Diseases*, *58*(SUPPL. 1), 10–19. https://doi.org/10.1093/cid/cit613

Tabor, D. E., Yu, L., Mok, H., Tkaczyk, C., Sellman, B. R., Wu, Y., … Esser, M. T. (2016). *Staphylococcus aureus* alpha-toxin Is conserved among diverse hospital respiratory isolates collected from a global surveillance study and is neutralized by monoclonal antibody MEDI4893. *Antimicrobial Agents and Chemotherapy*, *60*(9), 5312–5321. https://doi.org/10.1128/AAC.00357-16.Address

Tadesse, B. T., Ashley, E. A., Ongarello, S., Havumaki, J., Wijegoonewardena, M., González, I. J., & Dittrich, S. (2017). Antimicrobial resistance in Africa: A systematic review. *BMC Infectious Diseases*, *17*(1), 1–17. https://doi.org/10.1186/s12879-017-2713-1

Takeuchi, S., Ishiguro, K., Ikegami, M., Kaidoh, T., & Hayakawa, Y. (1998). Production of toxic shock syndrome toxin by *Staphylococcus aureus* isolated from mastitic cow's milk and farm bulk milk. *Veterinary Microbiology*, *59*(4), 251–258. https://doi.org/10.1016/S0378-1135(96)01253-9

Tavares, A., Nielsen, J. B., Boye, K., Rohde, S., Paulo, A. C., Westh, H., … Miragaia, M. (2014). Insights into Alpha-Hemolysin (Hla) evolution and expression among *Staphylococcus aureus* clones with hospital and community origin. *PLoS ONE*, *9*(7). https://doi.org/10.1371/journal.pone.0098634

Vubil, D., Garrine, M., Ruffing, U., Acácio, S., Sigaúque, B., Alonso, P. L., … Mandomando, I. (2017). Molecular characterization of Community Acquired *Staphylococcus aureus* bacteremia in young children in Southern Mozambique, 2001-2009. *Frontiers in Microbiology*, *8*(730), 1–8. https://doi.org/10.3389/fmicb.2017.00730

Wagenlehner, F. M. E., Schmiemann, G., Hoyme, U., Fünfstück, R., Hummers-Pradier, E., Kaase, M., … Naber, K. G. (2011). Nationale S3-Leitlinie .,Unkomplizierte Harnwegsinfektionen": Empfehlungen zu Therapie und Management unkomplizierter bakterieller ambulant erworbener Harnwegsinfektionen bei erwachsenen Patienten. *Urologe - Ausgabe A*, *50*(2), 153–169. https://doi.org/10.1007/s00120-011-2512-z

Woods, A., Couchman, J. R., Johansson, S., & Höök, M. (1986). Adhesion and cytoskeletal organisation of fibroblasts in response to fibronectin fragments. *The EMBO Journal*, *5*(4), 665–670. https://doi.org/10.1002/j.1460-2075.1986.tb04265.x

Zanger, P., Nurjadi, D., Schleucher, R., Scherbaum, H., Wolz, C., & Kremsner, P. G.

(2012). Import and spread of Panton-Valentine Leukocidin – Positive *Staphylococcus aureus* through nasal carriage and skin infections in travelers returning from the Tropics and Subtropics. *Clinical Infectious Diseases*, *54*(4), 483–492. https://doi.org/10.1093/cid/cir822

Zschöck, M., Botzler, D., Blöcher, S., Sommerhäuser, J., & Hamann, H. P. (2000). Detection of genes for enterotoxins (ent) and toxic shock syndrome toxin-1 (tst) in mammary isolates of *Staphylococcus aureus* by polymerase-chain-reaction. *International Dairy Journal*, *10*(8), 569–574. https://doi.org/10.1016/S0958-6946(00)00084-4

Acknowledgements

We thank the staff at Gambo General Rural Hospital, especially the laboratory workers, and the Microbiology Service at the Príncipe de Asturias University Hospital.

This work was partially funded by the University of Alcalá, and we also thank the other members of the University Group for Health Cooperation (UAH-GUdC16-02).

Figure Click here to access/download;Figure;figure.docx

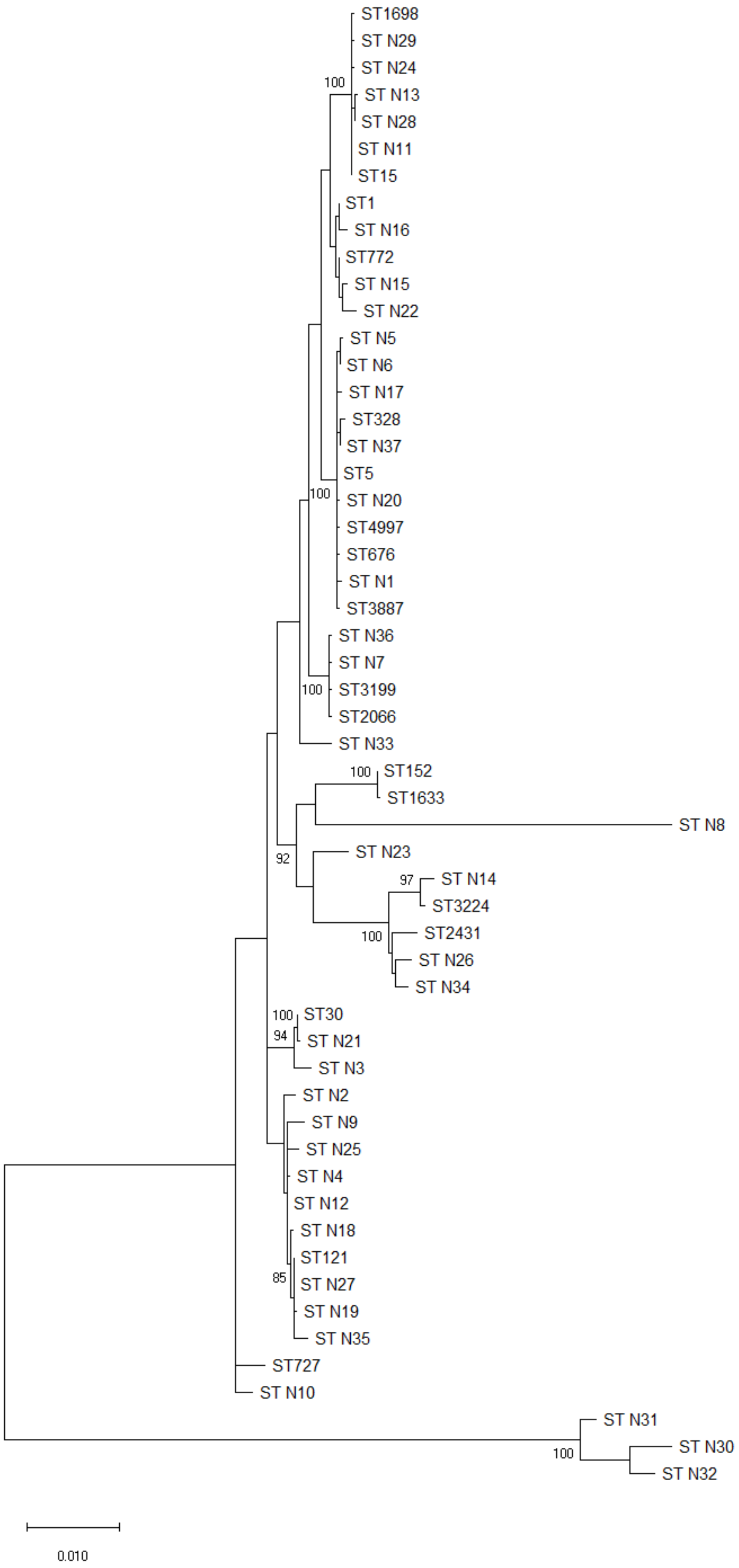

Table 1

Click here to access/download;Figure;table 1.docx

Antibiotic	Number of resistant (and intermediate) strains	% of resistant (and intermediate) strains	Antibiotic	Number of resistant (and intermediate) strains	% of resistant (and intermediate) strains
**P**	71	88.75	**Lvx**	1	1.25
**Am**	69 (1)	86.25 (1.25)	**Mxf**	1	1.25
**AUG**	3	3.75	**E**	15	18.75
**Ox**	3	3.75	**Cd**	23 (9)	28.75 (112.5)
**Cfxs**	1	1.25	**Syn**	0	0
**Cpt**	0	0	**Prs**	0	0
**Imp**	1	1.25	**Te**	42	52.5
**Gm**	12	15	**Min**	5	62.5
**To**	14	17.5	**Lzd**	0	0
**Ak**	15	18.75	**T/S**	4	50
**Va**	0	0	**Rif**	2	25
**Tei**	0	0	**Fos**	1	1.25
**Dap**	0	0	**Mup**	2	25
**Fd**	1	1.25	**C**	4	50
**Cp**	1	1.25			

Click here to access/download;Figure;table 2.docx

Strains	arc	aro	glp	gmk	pta	tpi	yqi	ST
Et	1	37	48	19	96	26	39	727
1	177	4	1	4	12	41	10	N1
2	6	5	6	2	7	14	5	121
3	6	5	6	2	7	14	5	121
4	6	22	6	2	7	14	10	N2
5	1	1	1	1	22	1	1	772
6	201	348	236	66	82	267	269	2431
7	2	4	N	3	144	3	2	N3
8	1	4	1	4	12	1	10	5
9	13	4	1	4	613	457	10	328
10	1	727	1	4	12	1	10	4997
11	13	13	1	1	12	11	13	15
12	6	5	6	2	7	14	655	N4
15	6	5	6	2	7	14	5	121
16	13	13	1	1	12	11	13	15
17	N	31	1	4	12	1	10	N5
18	13	13	1	1	12	11	13	15
19	46	75	49	44	13	68	60	152
20	13	13	1	1	12	11	13	15
21	46	75	49	44	13	68	60	152
22	N	4	1	4	12	1	10	N6
23	22	1	14	23	12	537	31	N7
24	6	1	298	44	289	291	N	N8
25	1	5	6	2	7	14	78	N9
26	10	349	6	2	260	58	2	N10
27	46	75	49	44	13	68	60	152
28	13	13	1	279	12	11	13	N11
29	13	13	1	279	12	11	13	N11
30	6	5	6	2	7	14	37	N12
31	46	75	212	44	13	68	60	1633
32	46	75	49	44	13	68	60	152
33	46	75	49	44	13	68	60	152
34	13	13	299	279	12	34	13	N13
35	354	256	358	281	221	302	13	N14
36	1	1	1	78	22	457	656	N15
37	1	1	1	279	67	486	656	N16
38	1	4	1	4	86	495	10	N17
39	6	5	6	281	7	14	94	N18
40	6	5	6	281	7	14	510	N19
41	1	4	1	4	12	1	256	N20
42	46	75	49	44	13	68	60	152
43	2	518	2	281	6	3	500	N21
44	6	1	1	279	22	457	541	N22
45	6	55	45	98	109	219	477	N23

**46**	6	5	6	281	7	14	94	N18
**47**	1	4	1	4	12	1	80	676
**48**	410	4	1	4	12	1	10	3887
**50**	13	643	1	279	12	11	13	N24
**51**	22	1	14	23	12	4	398	3199
**52**	354	256	358	66	221	302	328	3224
**53**	6	5	6	281	7	14	60	N25
**54**	354	N	236	169	194	411	N	N26
**55**	46	75	49	44	13	68	60	152
**56**	6	5	6	281	7	14	5	N27
**57**	13	13	299	279	12	11	13	N28
**58**	189	13	1	1	12	11	13	1698
**59**	6	5	6	281	7	14	5	N27
**61**	565	13	1	279	12	11	13	N29
**62**	7	6	1	8	8	8	6	N30
**63**	10	349	6	260	260	58	462	N31
**64**	1	4	1	12	12	1	10	N32
**65**	13	13	1	279	12	11	13	N11
**66**	46	75	49	44	13	68	60	152
**67**	46	75	49	44	13	68	60	152
**68**	22	1	14	23	12	4	233	2066
**69**	1	4	1	4	12	1	10	5
**70**	46	75	49	44	13	68	60	152
**71**	46	75	49	44	13	68	60	152
**72**	46	75	49	44	13	68	60	152
**73**	445	3	549	279	64	497	306	N33
**74**	6	5	6	281	7	14	5	N27
**75**	N	N	236	66	N	219	477	N34
**76**	46	75	49	44	13	68	60	152
**77**	46	75	49	44	13	68	60	152
**78**	6	5	6	281	7	48	5	N35
**79**	22	1	14	23	12	497	31	N36
**80**	1	4	1	4	12	457	10	N37
**81**	46	75	49	44	13	68	60	152
**82**	354	256	358	66	221	302	328	3224
**83**	13	13	1	279	12	11	13	N11
**84**	22	1	14	23	12	497	31	N36

Table 3 Click here to access/download;Figure;table 3.docx

	HLA	TST	PVL	FNBA	MECA
1	+	+	+	+	-
2	+	+	+	+	-
3	+	+	+	+	-
4	+	+	+	+	-
5	+	+	+	+	-
6	+	+	+	+	-
7	+	+	+	+	-
8	+	-	+	+	-
9	+	-	-	+	-
10	+	+	+	+	-
11	+	+	+	+	-
12	+	-	+	+	-
15	+	-	+	-	-
16	+	-	-	+	-
17	+	-	-	+	-
18	+	-	-	+	-
19	+	-	+	+	-
20	+	-	+	+	-
21	+	-	+	+	-
22	+	-	+	+	-
23	+	-	+	+	-
24	-	-	-	+	-
25	+	-	+	+	-
26	+	-	-	+	-
27	+	-	+	+	-
28	+	-	+	+	-
29	+	-	+	+	-
30	+	-	+	+	-
31	+	-	+	+	-
32	+	-	+	+	-
33	+	-	+	+	-
34	+	-	-	+	-
35	+	-	+	+	-
36	+	-	+	+	-
37	+	-	+	+	-
38	+	-	-	+	-
39	+	-	-	+	-
40	+	-	-	+	-
41	+	-	-	+	-
42	+	-	+	+	-
43	+	-	+	+	-
44	+	-	+	+	-
45	+	+	-	+	-

**46**	+	-	+	+	-
**47**	+	-	-	+	-
**48**	+	-	+	+	-
**50**	+	-	+	+	-
**51**	+	-	+	+	-
**52**	+	-	+	+	-
**53**	+	-	+	+	-
**54**	+	-	-	+	-
**55**	+	-	+	+	-
**56**	+	-	+	+	-
**57**	+	-	+	+	-
**58**	+	-	+	+	-
**59**	+	-	+	+	-
**61**	+	-	-	+	-
**62**	+	+	-	+	-
**63**	+	-	-	+	-
**64**	+	-	-	+	-
**65**	+	-	+	+	-
**66**	+	-	+	+	-
**67**	+	-	+	+	-
**68**	+	-	+	+	-
**69**	+	-	-	+	-
**70**	+	-	+	+	-
**71**	+	-	+	+	-
**72**	+	-	+	-	-
**73**	+	-	-	-	+
**74**	+	-	-	+	-
**75**	+	-	-	+	-
**76**	+	-	+	+	-
**77**	+	-	+	+	-
**78**	+	-	+	+	-
**79**	+	-	+	+	-
**80**	+	-	+	+	-
**81**	+	-	+	+	-
**82**	+	-	+	+	-
**83**	+	-	+	+	-
**84**	+	-	+	+	-

Click here to access/download
Supporting Information
Appendice.docx
