# Supplementary material for: Study of susceptibility to antibiotics and molecular characterization of high virulence Staphylococcus aureus strains isolated from a rural hospital in Ethiopia
Source: PLoS One. 2020 Mar 12;15(3):e0230031. doi: 10.1371/journal.pone.0230031 (PMC7067403; doi:10.1371/journal.pone.0230031)
Supplement: Attachment — Submitted filename: Response to Reviewers.docx [file pone.0230031.s006.docx]

Dear Reviewers and Editor,

Thank you for all your comments and suggestions. We have considered all your recommendations and we have modified the manuscript according with your indications.

Revisions and changes are marked in yellow, and parts deleted are marked with “strikethrough” in the “Revised Manuscript with Track Changes” document.

The manuscript format has been changed to follow the PLOS ONE’s style requirements.

Reviewer 1:

1. Please write the concentrations of the antibiotics and the name of the company which you purchased them.

The company of the used panel is indicated where says: “on a MicroScan®-Pos MIC Panel Type 33 (Beckman Coulter, B1016-173)”. Concentrations tested for each antibiotic are added, according with panel information that indicates in the website.

2. Please explain about the quality control of the susceptibility to antibiotic.

EUCAST rules have been follow and *S. aureus* strain 25923 ATCC is always used by the system as control and reference.

3. You said that, one of the most important virulence factors in S. aureus is the presence of antibiotic resistance, is that true? please mention the reference. In some rarely cases its right.

Although in some cases is considered as a virulence factors, it is truth that it is not a virulence factor in itself, so this clarification has been deleted, considering the antibiotic resistance an important factor in general in *S. aureus*.

4. Please explain about why the prevalence of MRSA is very low in your study in comparison to the other studiers in over the world which sated the prevalence of MRSA nearly over 40 %?

This is explained some sentences below, where say: “This difference may be due to the fact that our study was carried out in a rural area with predominantly community-acquired infections, whereas the other two studies were conducted in a crowded hospital using samples mainly collected from surgical wounds, suggesting hospital-acquired MRSA strains.”

Reviewer 2:

1. Number of strains that correspond to children and adults are indicated.
2. Synercid antibiotic name has been changed to “quinupristin/dalfopristin”.
3. Virulence genes are detected only by PCR, so methods are changed to describe this clearly.
4. Strains are always named as “strains”, not as “samples”
5. Discussion part referring to antibiotics susceptibility has been changed and genes information has been deleted in those cases is not necessary.
6. Mistakes have been corrected.
7. Acronyms for the names of antibiotics have been changed to 3 letters according with BSAC.
8. Table 2, referring to MLST typing results have been located in “supporting information” part and genes are written in italics as indicates in the bibliography, being necessary in some cases to use capital letters (for example gene *mecA*). “N” defined the new alleles and STs, as the text and the table heading describe.
9. Table 3 (now named 2) refers to amplificated genes in each strain, but description has been changed to be clearly.
10. Although there are a few studies about *Staphylococcus aureus* strains from Ethiopia, all of them are performed in cities and hospitals highly populated.
11. SCC*mec* typing as been determined and information of method and results have been added.
